# Lecithin-Linker Microemulsion Gelatin Gels for Extended Drug Delivery 

**DOI:** 10.3390/pharmaceutics4010104

**Published:** 2012-01-31

**Authors:** Xiao-Yue Xuan, Yu-Ling Cheng, Edgar Acosta

**Affiliations:** Department of Chemical Engineering and Applied Chemistry, University of Toronto, Toronto, ON M5S 3E5, Canada

**Keywords:** microemulsion-based gels, gelatin, lecithin, linker microemulsions, transdermal drug delivery

## Abstract

This article introduces the formulation of alcohol-free, lecithin microemulsion-based gels (MBGs) prepared with gelatin as gelling agent. The influence of oil, water, lecithin and hydrophilic and lipophilic additives (linkers) on the rheological properties and appearance of these gels was systematically explored using ternary phase diagrams. Clear MBGs were obtained in regions of single phase microemulsions (μEs) at room temperature. Increasing the water content in the formulation increased the elastic modulus of the gels, while increasing the oil content had the opposite effect. The hydrophilic additive (PEG-6-caprylic/capric glycerides) was shown to reduce the elastic modulus of gelatin gels, particularly at high temperatures. In contrast to anionic (AOT) μEs, the results suggest that in lecithin (nonionic) μEs, the introduction of gelatin “dehydrates” the μE. Finally, when the transdermal transport of lidocaine formulated in the parent μE and the resulting MBG were compared, only a minor retardation in the loading and release of lidocaine was observed.

## 1. Introduction

Transdermal drug delivery provides convenient and controlled delivery of drugs to patients with minimum discomfort [1]. The stratum corneum of the skin is the main barrier opposing transdermal absorption of drugs [2]. Microemulsions (μEs) have been proposed to overcome this barrier function and improve transdermal drug permeation [3,4,5,6,7,8,9,10,11]. Similar to transdermal delivery, periocular ophthalmic delivery is the least invasive method of delivery of drugs to the anterior and posterior section of the eye [12,13,14,15].The resistance to drug transport in periocular delivery is associated with the structure of corneal epithelium and stroma that act as protective barriers that regulate transport to and from the eye through the cornea and sclera [16]. Aqueous eye drops are the most popular dosage form in spite of their low bioavailability [17]. Eye drops formulated using μEs have been shown to improve the solubility of hydrophobic drugs and improve the efficacy of eye drop formulations [17,18,19]. 

Among the various μE systems, lecithin μEs are especially desirable since lecithin is a naturally occurring nontoxic biological surfactant with generally recognized as safe (GRAS) status [20]. However, lecithin cannot produce μEs when utilized as the sole surfactant because of its tendency to form liquid crystalline phases [6]. Earlier lecithin μEs were formulated using medium-chain alcohols, such as pentanol, that promote the μE phase [6]. Unfortunately, these medium-chain alcohols tend to dissolve cell membranes [21]. One alternative to the alcohol-based lecithin micromulsions is the use of linker molecules [22]. Alcohol-free lecithin μEs have been formulated with linkers as potential vehicles for transdermal drug delivery of lidocaine [22,23]. Linker molecules are amphiphilic additives that when added to the surfactant, tend to segregate near the surfactant tail (lipophilic linkers) or near the surfactant head group (hydrophilic linkers) [24]. The addition of linkers helps by increasing the surfactant-oil (lipophilic linkers) and surfactant-water (hydrophilic linkers) interactions. Furthermore, when hydrophilic and lipophilic linkers are combined, they produce a surfactant-like self-assembled system that offers enhanced solubilization capacity [25,26]. Compared to conventional alcohol-based lecithin μEs, lecithin-linker μEs have substantially lower toxicity, and can be prepared using food or pharmaceutical grade surfactants and linkers [22,23]. In addition, lecithin-linker μEs can provide twice the absorption and penetration of lidocaine through the skin when compared to conventional emulsions, and also demonstrated sustained transdermal delivery of lidocaine for 12 hours [22,23].

Compared to other pharmaceutical-grade μEs, lecithin-linker μEs have an exceptionally low viscosity (<150 mPa∙s) that, together with its affinity towards hydrophilic and lipophilic environments and small drop size (<10 nm), makes it a suitable vehicle to penetrate epithelial tissue and use these tissues as a depot for drug delivery [23,27]. While the low viscosity of lecithin-linker μEs is suitable for spray or roll-on methods of applications on the skin, these linker μEs spread well beyond the intended area when applied as drops [27]. The low viscosity of lecithin-linker μEs is also an undesirable feature in periocular ophthalmic delivery where higher viscosities are desirable to increase the residence time and improve the effectiveness of ophthalmic delivery formulations [28,29]. 

While the advantages and mechanisms of transdermal delivery with lecithin-linker μEs have been established in previous articles [22,23,27,30], the formulation objective for this work was to improve the rheological properties of these formulations, introducing gelatin as gelling agent, while retaining the desirable transport properties of the original (parent) μEs.

Under certain conditions, oil-continuous μEs can be transformed into highly viscous gels with the addition of certain gelling agents [31]. Since the main component is an organic solvent, these gels can be referred to as organogels [31]. A subgroup within the organogels is the μE-based gels (MBGs), introduced in the 1980s [32]. Generally, MBGs undergo liquid to gel transition when subjected to environmental stimuli such as changes in pH, temperature and electrolyte concentration [33]. Compared to their hydrogel counterparts, MBGs incorporate μE systems that provide a suitable environment for the solubilization of hydrophilic, lipophilic and amphiphilic drugs. Furthermore, due to their rheological properties, such as thermo-reversibility, MBGs have been proposed as potential vehicles for sustained drug and vaccine delivery [34], and as enzyme entrapment media (e.g., lipase) [35]. The gelling agents used to formulate these MBGs include natural polymers such as gelatin [36], κ-carrageenan [36], hydroxypropylmethyl cellulose (HPMC) [37], and block copolymer surfactants such as poly(ethylene oxide)-poly(propylene oxide)-poly(ethylene oxide), commercially known as Poloxamers or Pluronics [38]. Out of the many different MBGs reported, the majority have been prepared using gelatin with a Type II w/o micreomulsion system. Many of the reported μEs are systems of sodium dioctyl sulfosuccinate (AOT)/isooctane/water. Isopropyl myristate (IPM) has also been used as the oil phase. With the addition of 10 to 20% w/w solid gelatin, AOT μEs can be transformed into high viscosity, transparent thermo-reversible gels [31,32,33]. 

The gelation mechanism of gelatin-stabilized MBGs has been studied using various techniques, including small angle X-ray and neutron scattering (SAXS, SANS), ^1^H and ^13^C NMR, scanning emission and transmission electron microscopy (SEM, TEM), differential scanning calorimetry (DSC) and electrical conductometry [39]. Using small angle neutron scattering Atkinson *et al.* proposed a gelation model (for systems far from the phase transition point) where the gelatin strand network was formed in water-continuous channels and that the space in between the strands was filled by an oil-continuous AOT μE [40]. At the same time, for systems near the transition point towards the bicontinuous phase, Petit *et al.* proposed that only regions of the gelatin strands were formed in water-continuous environments, and that the rest of the strand, as well as the space in between the strands was filled with an oil-continuous AOT μE [41]. However, these groups agreed, and later studies confirmed, that in AOT μEs the introduction of gelatin had very little impact on the phase behavior of the microemulsion system [40,41,42]. 

A major limitation of gelatin MBGs is the toxicity associated with the anionic surfactant, AOT, used to formulate these μEs [43,44]. Gelatin MBGs prepared from lecithin μEs have been reported [36,37,45,46]. However, these lecithin MBGs contain short or medium chain alcohols and even the mildest formulation–produced with ethanol–still triggers some level of allergic response [46]. Willimann *et al.* formulated lecithin organogels (not classified as μEs by the authors) without alcohol or gelling agents and determined that these formulations were non-toxic and produced substantial trandermal permeation enhancement over conventional aqueous formulations [47]. However, the authors also indicated that it is necessary to use highly pure phosphatidyl choline to produce the gels and that the formulation is highly sensitive to the type of oil or drug used in the formulation. 

While gelatin MBGs have been used as reaction media [45,48,49], their use as drug delivery vehicles is limited since the presence of AOT and/or alcohols in these formulations poses cytotoxicity concerns. Recent articles on MBGs for transdermal delivery have concentrated on AOT-based MBGs, most of them using nonionic surfactants as additives [33,50,51,52]. Kantaria *et al.*, recognizing the biocompatibitlity issues of AOT, attempted to produce alternative MBGs formulated with nonionic surfactants but failed in producing these nonionic MBGs [50]. These authors linked this failure to the difference in the phase behavior of nonionic μEs compared to that of AOT (an anionic surfactant) systems. In one recent ion-sensitive nonionic MBG, formulated for periocular delivery of cyclosporine, the authors replaced water by glycerol in order to facilitate the formation of the gel [53]. That periocular delivery system (as well as many other ophthalmic formulations [54], including the formulation introduced in this work) made use of nonionic polyethylene glycol-based surfactants because of their biocompatibility. 

In the present study, we hypothesized that nonionic and alcohol-free gelatin-stabilized MBGs could be prepared using low toxicity linker-based lecithin μEs and that the increase in the viscosity of the formulation would not affect significantly the ability of the μE to permeate through epithelial tissue or membranes. To produce these lecithin-linker μEs, the formulation of Yuan *et al.* [22] was slightly modified by replacing the hydrophilic linkers sodium octanoate and octanoic acid with a milder combination of PEG-6-caprylic/capric glycerides and decaglycerol monocaprylate/caprate that has been confirmed to be non-irritant to human skin, non-mutagenic and highly biocompatible [27]. This base μE formulation has been used in the delivery of anti-wrinkle active ingredients in humans [27]. However, due to its low viscosity, these μEs spread beyond the intended area. 

As indicated by Kantaria *et al.* [50], the formulation of multicomponent nonionic MBGs is a complex task that requires careful consideration of the phase behavior of the μE and the properties of the resulting gel. Such consideration was lacking in the literature and was addressed in this study by comparing a set of phase behavior studies (phase scans and ternary phase diagrams) of the parent μEs with the appearance and rheological properties (particularly the elastic modulus, G’) of the resulting MBGs. Furthermore, the effect of each of the μE ingredients in the elastic modulus of the gels was evaluated in order to determine the relative importance of each ingredient in the rheology of the gels. 

To evaluate the effect of gelatin on drug transport through epithelial tissue, the release profiles of lidocaine, topically adsorbed in the skin from a lecithin-linker gelatin MBG and from the parent μE, were obtained. To evaluate the effect of gelatin on transmembrane transport properties, a membrane permeation study was conducted to compare the permeation profiles of lidocaine incorporated in a gelatin MBG and in the parent μE, using conditions relevant to periocular delivery. 

## 2. Materials and Methods

### 2.1. Materials

#### 2.1.1. Chemicals

The following chemicals were purchased from Sigma-Aldrich (Oakville, ON, Canada) and were used as received: gelatin (from porcine skin, type A, 300 g Bloom), isopropyl myristate (IPM, 98%), sorbitan monooleate (SMO, Span^®^ 80, 99%), sodium chloride (99%+, Fluka brand), Dulbecco’s phosphate buffered saline (PBS) and lidocaine powder (base form, 98%+). Decaglycerol monocaprylate/caprate (Drewpol 10-1-CC, 97%) was a gift from Stepan Company (Northfield, IL, USA) and PEG-6-caprylic/capric glycerides (Softigen 767, 98%+) was donated by Sasol North America (Houston, TX, USA). Laboratory grade soybean lecithin was purchased from Fisher Scientific (Fairlawn, NJ, USA). Soybean lecithin (99% phospholipids) was a mixture of phospholipid (mainly phosphatidylcholines) obtained by acetone purification of soybean gum residues. The average composition of soybean lecithin has been reported elsewhere [55]. Acetonitrile (HPLC grade) was purchased from Caledon Laboratories Ltd. (Georgetown, ON, Canada), sodium phosphate monobasic, monohydrate (ACS grade) was purchased from EMD Chemicals Inc. (Darmstadt, Germany), and were used as received. Anhydrous ethyl alcohol was purchased from Commercial Alcohols Inc. (Brampton, ON, Canada). The composition was expressed on a mass basis (*i.e.*, % w/w.) throughout this work.

#### 2.1.2. Skin

Ear skin from domestic pigs (approximately 6 months old) was used as a surrogate for human epidermis [56]. Porcine ears were purchased from a local market and frozen overnight. They were partially thawed by rinsing with running water for 10 seconds at room temperature to soften the skin for smoother cuts. The skin of the external side of the ear was dermatomed to a thickness ranging from 700 to 900 μm [57]. Areas of the skin with cuts, burns, swelling or abnormal texture were avoided. The dermatomed tissue was cut into circles of 11.4 mm diameter. Before placing the skin samples in the permeation device, the samples were equilibrated to room temperature and then inspected to make sure that there were no pores or other imperfections. The work of Yuan *et al.* includes more details about pig skin sampling and its use to evaluate the transdermal permeation in linker μEs [22,23,30].

### 2.2. Microemulsion (μE) Preparation

μEs were prepared in flat-bottom tubes by mixing lecithin (surfactant), decaglycerol monocaprylate/caprate and PEG-6-caprylic/capric glycerides (hydrophilic linkers), sorbitan monooleate (SMO, lipophilic linker), isopropyl myristate (IPM) and 0.9% sodium chloride solution at various compositions (Table 1). The concentration of lecithin, PEG-6-caprylic/capric glycerides and decaglycerol monocaprylate/caprate was kept at 5% w/w, 10% w/w and 4% w/w, respectively (giving a 1:2:0.8 weight ratio of lecithin to PEG-6-caprylic/capric glycerides to decaglycerol monocaprylate/caprate). This might not be the optimal composition but it was the maximum ratio of lecithin to hydrophilic linkers that could be used to produce μE systems without the formation of meta-stable phases. In addition, a minimum of 2% SMO was also required to prevent these meta-stable phases. The phase scan of the μE consisted of increasing SMO concentration from 2% to 10% (g SMO/g total formulation), thus increasing the hydrophobicity of the formulation. After introducing all the ingredients, the tubes were thoroughly vortexed and left to equilibrate for 2 weeks.

**Table 1:** Composition of lecithin-linker microemulsion phase scans.

Component	Composition (% w/w)
0.9% sodium chloride in de-ionized water	36
PEG-6-caprylic/capric glycerides	10
Decaglycerol monocaprylate/caprate	4
Lecithin	5
Sorbitan monooleate (SMO)	2-10
Isopropyl myristate (IPM)	To 100%

### 2.3. MBG Preparation

MBGs were prepared by addition of gelatin powder to o/w μEs at various surfactant/linker/oil/water ratios, according to the method of Kantaria *et al.* [33]. Briefly, the mixture was first stirred at room temperature for 45 minutes to swell the solid gelatin, and then heated to 50 °C and stirred for 20 minutes until the gelatin was completely dissolved in the μE. The agitation was then stopped and the sample was allowed to cool in an ice bath for 30 minutes. MBGs with various hardness and opacity were obtained.

### 2.4. Ternary Phase Diagrams

Phase behaviour studies were performed by constructing ternary phase diagrams at room temperature (25 °C) and at the gelatin activation temperature (50 °C) using the water titration method [58,59]. The “surfactant” vertex of the ternary phase diagrams was a mixture of 1:2:0.8:1.2 weight ratio of lecithin: PEG-6-caprylic/capric glycerides:decaglycerol monocaprylate/caprate:SMO. Mixtures of lecithin + linkers and isopropyl myristate were prepared; then an aqueous solution of 0.9% sodium chloride solution was added until the desired composition along the dilution line was achieved. After each titration the flat-bottom tubes were vortexed for 3 minutes to ensure thorough mixing. The phase behavior of each of the tubes at room temperature was observed after two weeks of equilibration time. The ternary phase diagram at 50 °C was generated by keeping the tubes in a constant-temperature water bath at the desired temperature for two weeks, which allowed the systems to reach the new equilibrium. Anisotropic liquid crystal (LC) phases in the phase diagrams were identified using cross polarization microscopy with an Olympus BX-51 microscope (Richmond Hill, ON, Canada). Precipitate (P) phases were identified as systems where a solid residue was observed at the bottom of the test tube. Microemulsion phases were identified as translucent yellow-amber phases—this color was indicative of the presence of lecithin, SMO and the hydrophilic linkers in that phase—that were able to scatter (but not diffuse) a red laser beam (650 nm). Further confirmation of the microemulsion phase (μE) was obtained via electrical conductivity measurements and via repeated temperature cycles (25 °C–50 °C–25 °C) where the microemulsion phase volumes were recovered after completing each cycle. The presence of excess oil (Oil) phase or aqueous (Water) phases was confirmed via electric conductivity measurements. It is important to note, however, that these “Oil” or “Water” phases contained part of the linkers that partitioned into these excess phases. Another way of differentiating μEs from the “Oil” and “Water” phases was that the latter were not able to scatter the light of the red laser beam. The characteristics of the surfactant “S” phase identified in the systems containing precipitate (P) were similar to those of a μE system, only that this S phase had a lighter amber colour, and produced a weaker scattering of the red laser beam than its μE counterpart. The composition or structure of the S, LC, P, Oil and Water phases were not further studied because they were not useful in the preparation of lecithin- linker MBGs for transdermal delivery. 

### 2.5. Physiochemical Characterization

The conductivity of the μEs was measured at room temperature using a VWR bench/portable conductivity meter equipped with a custom-build OEM conductivity microelectrode (Microelectrodes Inc., Bedford, NH, USA). Viscosity measurements of the μE samples were obtained (in triplicate) using a CV-2200 falling ball viscometer (Gilmont Instruments, Barrington, IL, USA) at room temperature. The hydrodynamic diameter measurements of the μE aggregates were determined via dynamic light scattering at a 90° angle, using a BI 90Plus particle size analyzer equipped with a 35 mW diode laser (wavelength ~674 nm) (Brookhaven Instruments, Holtsville, NY, USA).

### 2.6. Rheological Characterization

Rheological characterizations of the MBGs were performed using a stress and strain controlled CSL^2^ 500 rheometer (TA Instruments Ltd., Surrey, UK). The measuring system used was the 4 mm diameter stainless steel cone and plate geometry (cone angle 2°). The sample volume was approximately 1 mL. Oscillation experiments were performed at 1.00 Hz frequency and 0.177 Pa oscillation stress over the temperature range of 20 to 50 °C. At the maximum temperature the formulations were liquid and then gelled upon cooling. The elastic modulus G’ was recorded. 

### 2.7. *In Vitro* Transport of Lidocaine

To evaluate the performance of gelatin-stabilized MBGs as delivery vehicles, a lipophilic drug, lidocaine base, was chosen as the model drug in this work. Lidocaine is an anesthetic used in topical formulations as a pain reliever in the treatment of minor burns, after various laser skin surgeries and during cataract surgery [60,61,62]. It was incorporated in the lecithin-linker μEs and gelatin MBGs by pre-dissolving 10% w/w lidocaine in isopropyl myristate (IPM). The methods described below were adapted from previous transdermal transport studies for lidocaine, formulated in lecithin-linker μEs [22,23,30]. 

#### 2.7.1. Transport of Lidocaine in the Skin

The transdermal *in vitro* extended release experiments for a selected μE and its gelatin MBG were conducted using MatTek Permeation Device (MPD) supplied by MatTek Corporation (Ashland, MA, USA) fitted with pig ear skin as membrane. The experiment was carried out in laboratory conditions that simulated the topical application of these formulations. Briefly, pig ear skin tissues were placed into the MPD with the epidermis layer facing up. The exposed tissue area in the MPD was 0.256 cm^2^. After assembling the device, 400 μL of test μE (25 °C) or liquefied gelatin MBG (initially at 50 °C) were applied in the donor compartment. During the transfer process, the liquid gelatin-μE mixture was cooled down to 40 °C or less, making it unlikely for the MBG to affect the structure and permeability of the stratum corneum. The receptor compartment was filled with 5 mL of PBS. The donor μE or the gelatin MBG was withdrawn 30 minutes after application [22,23]. The skin surface in the MPD was blotted dry with Kimwipes and was then used for extended release. At predetermined times (1, 3, 6, 12, 24 and 48 h), the receiver solution was withdrawn completely from the receptor compartment and was immediately replaced with fresh PBS solution to maintain sink conditions. The experiment was terminated at 48 h. At the end of the experiment, the pig ear skin from each of the MPD was collected and used to determine the final concentration of lidocaine absorbed in the skin (and hence the total amount of lidocained absorbed in the skin). To this end, the skin samples were rinsed with a few droplets of PBS solution and then the residual lidocaine in each sample was extracted with 2 mL of methanol for 48 h [22,23]. All experiments were conducted in quadruplets at room temperature.

#### 2.7.2. Transmembrane Transport of Lidocaine

*In vitro* permeation experiments were conducted as described in Section 2.7.1 with four modifications: (A) the skin tissue was replaced with cellulose acetate membranes (Harvard Apparatus, MWCO 100 kDa, ~10 nm pore size); (B) An aliquot of 50 µL of test μE (25 °C) or liquefied MBG (50 °C) was applied to the donor compartment and was kept in the donor compartment throughout the 48 h permeation study (instead of removing it after 30 minutes of loading); (C) The receptor compartment was filled with 5 mL of simulated tear fluid (Table 2) instead of PBS; (D) All permeation experiments were conducted in quadruplets at 34 °C (ocular surface temperature). These modifications were introduced to simulate the release from the MBG and its corresponding μE onto a solution that simulates tear fluid as a simple model for periocular delivery.

**Table 2:** Simulated tear fluid composition (receiver solution in transmembrane transport studies) [63].

CaCl_2_ ∙ 2H_2_O NaCl H_2_O (pH 7.2)	0.008 g 0.658 g To 100 g

### 2.8. Lidocaine Quantification

The concentration of lidocaine in the skin.—determined after extraction with methanol.—and in receiver solutions, was quantified using a Dionex ICS-3000 liquid chromatography system equipped with an AS40 automated sampler, AD25 absorbance detector and a reverse phase column (Genesis, C_18_, 4 μm, 150 mm × 4.6 mm). The UV detector was set to 230 nm. A mixture of acetonitrile and 0.05M NaH_2_PO_4_∙H_2_O (pH 2.0) (30:70, v/v) was used as the mobile phase at flow rate of 1.0 mL/min. The retention time of lidocaine under the described conditions was approximately 2.7 min, and the calibration curve for the area under the peak *vs.* concentration was linear (R^2^ = 0.9997). Further details of the development and validation of this liquid chromatography method can be found in the work of Yuan *et al.* [22].

## 3. Results and Discussion

### 3.1. Phase Behaviour of Lecithin-Linker Microemulsions (μEs)

#### 3.1.1. Sorbitan Monooleate (SMO) Phase Scan

**Figure 1:**
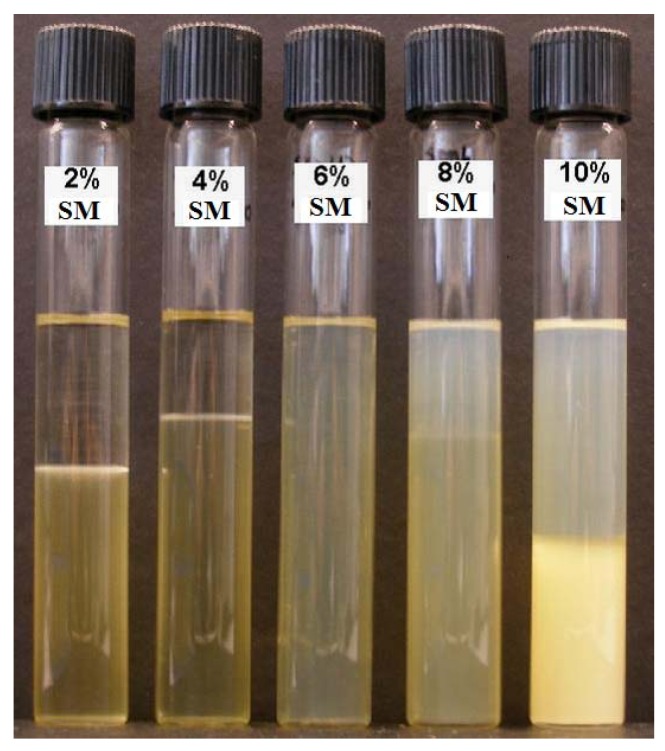
Phase scan at room temperature (25 ± 1 °C). All test tubes contained 5% lecithin, 10% PEG-6 caprylic/capric glycerides and 4% decaglycerol monocaprylate/caprate (see Table 1 for additional composition details).

The compositions of linker-based lecithin μEs considered in the SMO scan are shown in Table 1. A picture of the vials employed in this phase scan is presented in Figure 1, showing the transition from Type I o/w μE (bottom phase) with excess oil phase (top phase) obtained at 2% and 4% SMO to single phase Type IV μE at 6% SMO. The systems of 8% and 10% SMO produced a more complex behaviour that included a top phase oil-continuous (Type II) μE. The types of μEs produced were confirmed using conductivity measurements (Table 3). 

**Table 3:** Properties of the μEs produced with the phase scan (2–10% sorbitol monooleate—SMO) of Figure 1 (see Table 1 for additional composition details) at room temperature (25 ± 1 °C).

% SMO	2%	4%	6%	8%	10%
Conductivity (μS/cm) of microemulsions (μEs) and excess phases					
**Top**	0.0 ± 0.0	0.0 ± 0.0	1500 ± 11	40.3 ± 1.5	30.7 ± 2.1
**Bottom**	1660 ± 10	1600 ± 10		1320 ± 6	1220 ± 6
Hydrodynamic radii (nm) of μEs					
	1.7 ± 1	5.9 ± 3.9	2.5 ± 1.6	N.M.	N.M.
Viscosity (mPa∙s) of μEs					
	28.3 ± 0.0	63.2 ± 0.5	126.8 ± 2.7	N.M	N.M

N.M.: values not measured.

Yuan *et al.* evaluated Type I, IV and II lecithin linker μEs as lidocaine delivery vehicles and found that while all these types improved lidocaine loading in the skin and its transdermal flux, Type IV systems produced the largest lidocaine loading in the skin [22]. Furthermore, bicontinuous (Type IV) μEs produce the largest co-solubilization of oil and water. Considering the advantages of Type IV formulations, the 6% SMO system was selected as the base composition to construct ternary phase diagrams and to evaluate *in vitro* transport of lidocaine. 

The viscosity of μE formulations containing 2 to 6% SMO are presented in Table 3. The increase in viscosity from 28 to 127 mPa∙s with increasing SMO concentration can be explained on the basis that when approaching the Type I-IV transition (increasing SMO), oil-swollen micelles grow larger and turn cylindrical, which increases the viscosity of the formulation [22,64]. The data in Table 3 confirms the relatively low viscosity of lecithin-linker μEs when compared to medium-chain alcohol lecithin μEs that reach viscosities as high as 1000 mPa∙s [31] and to commercial topical creams such as Lanocort 10 that have viscosities of approximately 1300 mPa∙s [65]. The low viscosity of lecithin-linker μEs makes them suitable for spray and roll-on applications, but it is a disadvantage for gel-type topical and ophthalmic applications.

The mean hydrodynamic radii, obtained via dynamic light scattering, for the μEs of Figure 1 containing 2%, 4% and 6% SMO are also presented in Table 3. These radii are comparable to the values reported by Yuan *et al.* for similar linker-lecithin systems [22]. The small droplet size (less than 10 nm) of lecithin-linker μEs has been associated with the use of hydrophilic linkers that increase the interfacial area and reduce the size of oil-swollen micelles and water-swollen reverse micelles [25,66]. Although the hydrodynamic radius for 6% SMO is also reported in Table 3, the meaning of that particular measurement is questionable because the μE does not exist as disperse droplets but as interconnected (bicontinuous) channels. 

#### 3.1.2. Ternary Phase Diagrams

**Figure 2:**
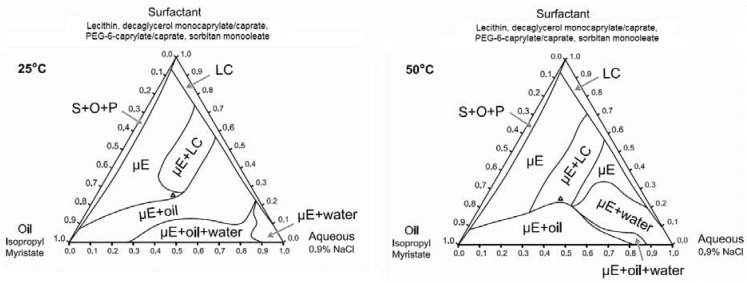
Ternary phase diagrams of 6% SMO formulation at 25 °C and 50 °C. The surfactant vertex is a mixture of 1:2:0.8:1.2 weight ratio of lecithin: PEG-6-caprylic/capric glycerides:decaglycerol monocaprylate/caprate:SMO. The point “∆” indicates the composition of the base formulation containing 6% SMO.

Ternary phase diagrams at 25 °C and 50 °C (gel activation temperature) were constructed using the surfactant composition corresponding to the 6% SMO formulation of Figure 1. These phase diagrams are presented in Figure 2. At both temperatures, the surfactant mixture was not completely soluble in either the aqueous (0.9% NaCl) solution or in isopropyl myristate (IPM). Liquid crystalline (LC) or surfactant + oil + precipitate phases (S + O + P) were found in systems containing less than 10% IPM or 5% water. Other phases observed in the ternary phase diagrams include isotropic, single phase microemulsions (µE), μE in equilibrium with dispersed liquid crystalline phases (µE + LC), μEs in equilibrium with excess oil (µE + oil), μE in equilibrium with excess water (µE + water), and μE coexisting with excess oil and excess water phases (µE + oil + water). The main difference in the ternary phase diagram at 50 °C compared to that at 25 °C is the larger µE+water region at 50 °C. This can be explained on the basis that the hydrophilic linker PEG-6-caprylic/capric glycerides are temperature sensitive, due to the presence of ethylene oxide groups [67]. As the temperature increases from 25 °C to 50 °C, hydrogen bonding between the ethylene oxide groups of the hydrophilic linkers and the water molecules weaken (dehydrate) and the formulations become more hydrophobic, hence the ability of the μE to solubilize water decreases, resulting in the formation of a larger µE + water region [67,68,69].

### 3.2. Phase Behaviour of Lecithin-Linker MBGs

#### 3.2.1. Sorbitan Monooleate (SMO) Phase Scan

**Figure 3:**
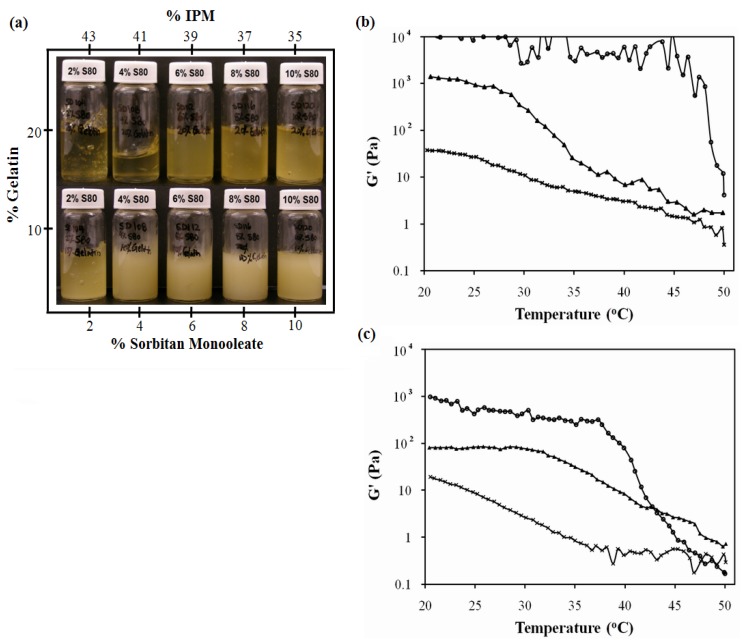
SMO scan of lecithin-linker MBGs. All formulations contain 10% PEG-6-caprylic/capric glycerides, 4% decaglycerol monocaprylate/caprate, 5% lecithin, 36% of 0.9% NaCl solution and the balance of isopropyl myristate (IPM). (**a**) Gel appearance. Elastic modulus (G’) *vs.* temperature profile of (**b**) 20% gelatin MBGs and (**c**) 10% gelatin MBGs containing (×) 2% SMO; (▲) 10% SMO; Control (10% or 20% gelatin in 0.9% NaCl solution) (●) Table 1 present additional μE composition details. For the systems of panel (**b**), a linear regression between log(G’) and T (from 25 to 40 °C) was evaluated for gelatin, log(G’,Pa) = 4.7(±0.3) − 0.027(±0.009)T(°C), (R^2^ = 0.27); for 10% SMO-gelatin log(G’,Pa) = 7.3(±0.2) − 0.163 (±0.009)T(°C), (R^2^ = 0.98); for 2% SMO-gelatin log(G’,Pa) = 2.93(±0.06) − 0.062 (±0.002)T(°C), (R^2^ = 0.97). The ±deviations in parentheses represent the standard deviation of the regression parameters (Excel 2010 data analysis pack).

The systems of Figure 1 were used to produce MBGs formulated with 10% and 20% gelatin. Figure 3a presents pictures of these MBGs. MBGs formulated with 2%, 4% and 6% SMO and 20% gelatin were translucent. The elastic modulus (G’) of MBGs produced with 2% and 10% SMO are presented in Figure 3b and 3c as a function of temperature for formulations containing 10% and 20% gelatin, respectively. Higher G’ values were obtained in systems formulated with 20% gelatin, indicating the formation of stronger gels, likely due to a denser gel network structure. Furthermore, gels with lower SMO produced weaker, albeit more clear, gels.

The electrical conductivity of the clear gels produced with 2–6% SMO ranged from 50 to 200 μS/cm, suggesting that although the original μEs were water-continuous systems, the structure of the final gel was closer to a bicontinuous μE rich in oil. The fact that oil-rich bicontinuous MBGs were produced is consistent with the work of Atkinson *et al.* and Petit *et al.* on AOT MBGs [40,41]. However, in contrast with all the work reported on AOT-based MBGs, where the addition of gelatin does not affect the phase behavior of the parent μE, the phase behavior of the lecithin-linker (nonionic) systems was almost reversed with the addition of 20% gelatin. One explanation for the fact that the presence of gelatin induced the transition from Type I μEs to bicontinuous oil-rich systems, is that gelatin “dehydrated” the μE and used that water to form the gel network. According to the ternary phase diagrams of Figure 2, removing water from the μE shifts the formulation into a region of single phase μEs rich in oil. For the MBGs containing 8 and 10% SMO, their marginally translucent appearance might be a sign that the final μE phase in the MBG is closer to a phase transition.

**Figure 4:**
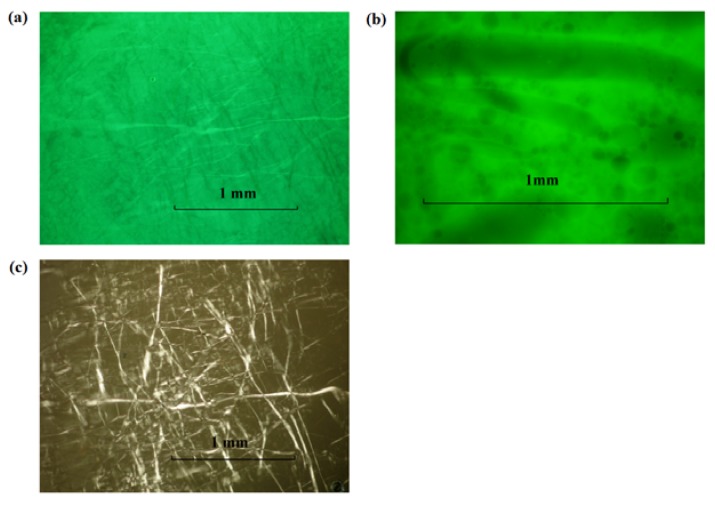
Fluorescence image of lecithin-linker 2% SMO MBG (20% gelatin) at room temperature labeled with (**a**) sodium fluorescein and (**b**) nile red; (**c**) Cross-polarized light image of the sample of panel (**a**). For formulation details see Figure 1 and Table 1.

**Figure 5:**
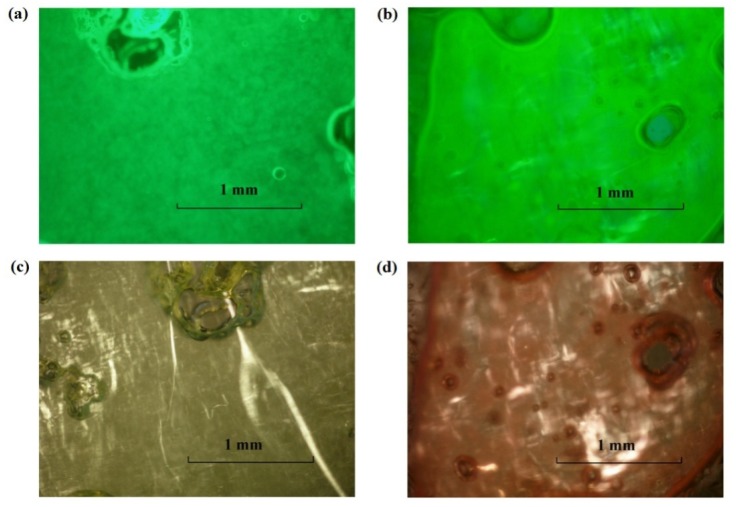
Fluorescence image of lecithin-linker MBG (20% gelatin) with 10% SMO labelled with (**a**) Sodium Fluorescein and (**b**) Nile Red. Cross-polarized light images of lecithin-linker MBG of (**c**) the sample of panel (**a**) and (**d**) the sample of panel (**b**). For formulation details see Figure 1 and Table 1.

Fluorescent dyes, sodium fluorescein (hydrophilic) and nile red (hydrophobic), were added (separately) to the 2% and 10% SMO MBGs produced with 20% gelatin to assess macroscopic phase separation and the structure of the gel fibers, using florescence and polarized light microscopy. According to Figure 4, the MBG formulated with 2% SMO showed apparent continuity in both aqueous (4a) and oil (4b) phases according to the continuous green color from fluorescein (water soluble) and nile red (oil soluble), respectively. Areas that showed different color intensity were typically indicative of bubbles or gelatin fibers in the sample. The structure of these fibers was evidenced by the bright strands observed under cross polarizers in Figure 4c. Similarly, the MBG containing 10% SMO also seemed to be continuous in both phases (Figure 5), and also had a three-dimensional network of gelatin fibers. It is important to clarify that at the magnification scale of Figure 4 and Figure 5, it was not possible to assess the continuity of these systems because of the submicron scale of the μE domains. However, the macroscopic observations with the fluorescence dyes were consistent with the electrical conductivity measurements in the gels discussed earlier.

According to Figure 5c and Figure 5d, the fibers of the 10% SMO MBG were thicker than those shown in Figure 4c for 2% SMO MBG. This difference in fiber thickness could explain the higher gel modulus (G’) and turbidity of MBGs prepared with 10% SMO.

#### 3.2.2. Ternary Phase Diagrams

Gel formation in gelatin MBGs occurred between room temperature (25 °C) and 50 °C. At 50 °C, the gelatin activation temperature, the native helical structure of gelatin is denatured, and collagen exists as flexible random coils in solution [70]. Upon cooling, gelatin recovers its native helical structure, producing a gelatin hydrogel network [70]. Using the ternary phase diagrams of Figure 2, the appearance and rheological properties of MBGs, along oil and water dilution lines that passed through the optimal 6% sorbitan formulation (containing 36% water and 39% oil), were evaluated.

**Figure 6:**
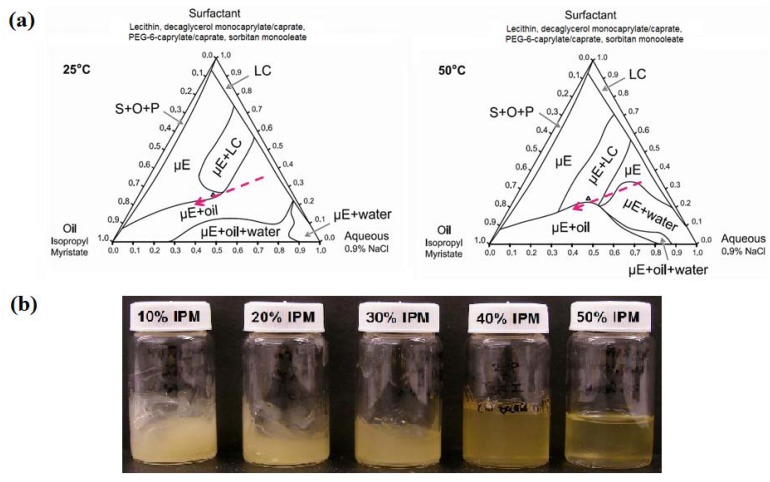
(**a**) Oil dilution path (dashed arrow) used to produce MBGs with 10% to 50% IPM; (**b**) Appearance of MBGs at room temperature. Ternary phase diagrams at 25 °C and 50 °C. The surfactant vertex is a mixture of 1:2:0.8:1.2 weight ratio of lecithin: PEG-6-caprylic/capric glycerides:decaglycerol monocaprylate/caprate:SMO.

Figure 6a shows the oil dilution path at 25 °C and 50 °C. Figure 6b presents a picture of the gels prepared along the oil dilution line. For systems containing between 10% and 30% IPM, the gels had a *milky* appearance, suggesting the presence of multiple phases in the gel. Considering the earlier discussion that gelatin “dehydrated” the original μE, and the ternary phase diagrams of Figure 6a, it is likely that the gelling process for these 10–30% IPM systems led to the formation of a μE + LC systems embedded in the gelatin hydrogel. Although the system containing 50% IPM was clear, it was a 2-phase system of μE and a MBG. The 40% IPM formulation was close to the 6% SMO gel of Figure 3a. 

**Figure 7:**
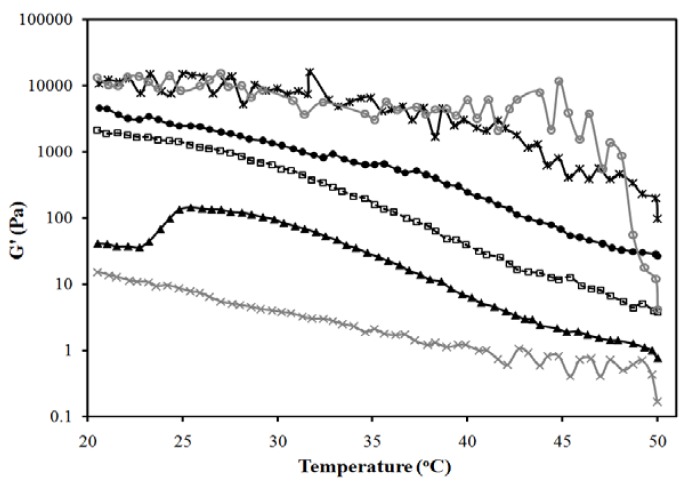
Elastic modulus *vs.* temperature profile for the systems of Figure 6b: (●) 10% IPM; (□) 20% IPM; (▲) 30% IPM; (×) 40% IPM; (*) 50% IPM. Control (20% gelatin in 0.9% NaCl solution) is shown as (○). Composition given in Figure 6a, following the oil dilution line (dash line). A linear regression between log(G’) and T (from 25 to 40 °C) was evaluated for gelatin, log(G’,Pa) = 4.7(±0.3) − 0.027(±0.009)T(°C), (R^2^ = 0.27); for 10% IPM log(G’,Pa) = 5.11(±0.07) − 0.068 (±0.002)T(°C), (R^2^ = 0.97); for 20% IPM log(G’,Pa) = 6.02(±0.08) − 0.111 (±0.002)T(°C), (R^2^ = 0.99); for 30% IPM log(G’,Pa) = 4.8(±0.1) − 0.099 (±0.003)T(°C), (R^2^ = 0.97); for 40% IPM log(G’,Pa) = 2.27(±0.05) − 0.057 (±0.001)T(°C), (R^2^ = 0.98); for 50% IPM log(G’,Pa) = 5.12(±0.17) − 0.042 (±0.005)T(°C), (R^2^ = 0.72). The ±deviations in parentheses represent the standard deviation of the regression parameters (Excel 2010 data analysis pack).

Figure 7 presents the temperature dependence of the elastic modulus (G’) of the gels of Figure 6b. According to Figure 7, increasing the oil (IPM) content in the MBG from 10 to 40% reduces the strength of the gel, albeit increasing its clarity. To obtain a measurement for the 50% IPM MBG, only the gel portion of the 2-phase system was evaluated, which explains the high strength of this gel.

For the 40% IPM linker-lecithin MBG, the zero shear viscosity was close to 3 Pa∙s at 25 °C and 1 Pa∙s at 37 °C, which represents one order of magnitude increase in viscosity with respect to the original μE. Viscosities in the 1–10 Pa∙s range are comparable to some topical creams [65]. The gel strength of the system with 40% IPM is lower than other commercial lidocaine creams and gels (measured G’ at 25 °C for EMLA^®^ cream and Topicaine gel were approximately 500 Pa and 200 Pa, respectively). As shown in Figure 3, MBGs with high G’ values can be obtained with higher gelatin and SMO content.

**Figure 8:**
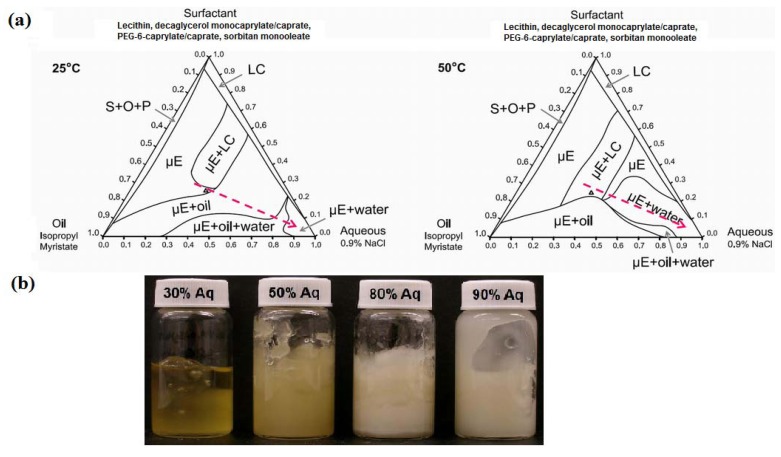
(**a**) Water dilution path (dashed line) of MBGs containing 30% to 90% water; (**b**) MBG appearance at room temperature. Ternary phase diagrams at 25 °C and 50 °C. The surfactant vertex is a mixture of 1:2:0.8:1.2 weight ratio of lecithin: PEG-6-caprylic/capric glycerides:decaglycerol monocaprylate/caprate:SMO.

**Figure 9:**
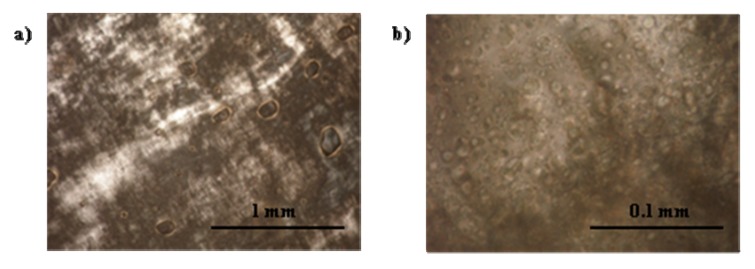
Cross-polarizer micrographs of 20% gelatin MBG formulated with a μE containing 80% water (system of Figure 8b), 10% oil and a 10% of a mixture of 1:2:0.8:1.2 weight ratio of lecithin: PEG-6-caprylic/capric glycerides:decaglycerol monocaprylate/caprate:SMO.

Figure 8a presents the water dilution path used to produce MBGs containing 30 to 90% water. Figure 8b shows that with increasing water content the MBG became more turbid. These milky gels reflect the presence of an emulsified phase within the gel. These gel-entrapped emulsions were likely produced when, at 50 °C, a μE phase coexisted with an aqueous solution, as shown in Figure 8a. The gelatin gel was probably formed within the excess aqueous phase and, upon cooling, the μE and any excess water—not associated with gelatin—were emulsified within the gel. Figure 9 shows cross-polarizer micrographs of the MBG prepared with 80% water, showing the gelatin network (Figure 9a) and the drops of the emulsion entrapped in the gel (Figure 9b). 

**Figure 10:**
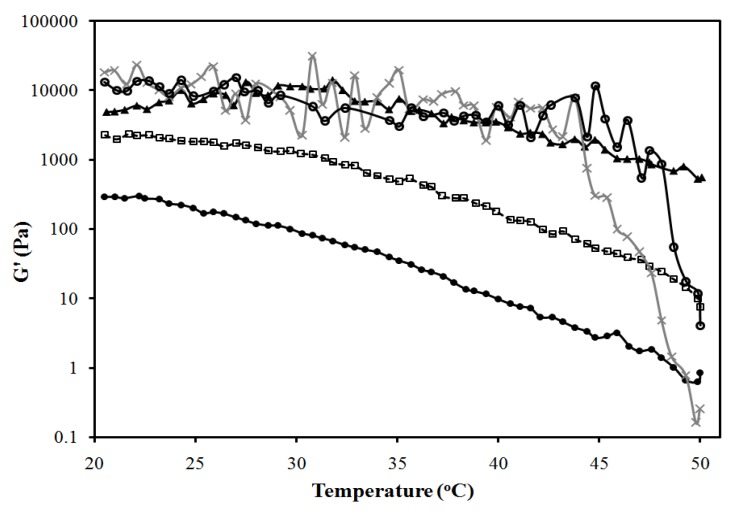
Elastic modulus *vs.* temperature profile for the systems of Figure 8b: (▲) 30% aqueous; (●) 50% aqueous; (□) 80% aqueous; (×) 90% aqueous. Control (20% gelatin in 0.9% NaCl solution) is shown as (○). A linear regression between log(G’) and T (from 25 to 40°C) was evaluated for gelatin, log(G’,Pa) = 4.7(±0.3) − 0.027(±0.009)T(°C), (R^2^ = 0.27); for 30% water log(G’,Pa) = 4.78(±0.21) − 0.031 (±0.006)T(°C), (R^2^ = 0.52); for 50% water log(G’,Pa) = 4.52(±0.07) − 0.087 (±0.002)T(°C), (R^2^ = 0.98); for 80% water log(G’,Pa) = 5.24(±0.09) − 0.074 (±0.003)T(°C), (R^2^ = 0.96); for 90% water log(G’,Pa) = 4.51(±0.37) − 0.019 (±0.011)T(°C), (R^2^ = 0.104). The ±deviations in parentheses represent the standard deviation of the regression parameters (Excel 2010 data analysis pack).

Figure 10 presents the elastic modulus (G’) for the MBGs of Figure 8b, as a function of temperature. In general, increasing the water content in the MBGs increases the strength of the gels. However, the system containing 30% water, similar to the 50% oil MBG, consists of a liquid oil-rich μE that coexists with a strong gel. 

#### 3.2.3. Lecithin-Linker Gels

**Figure 11:**
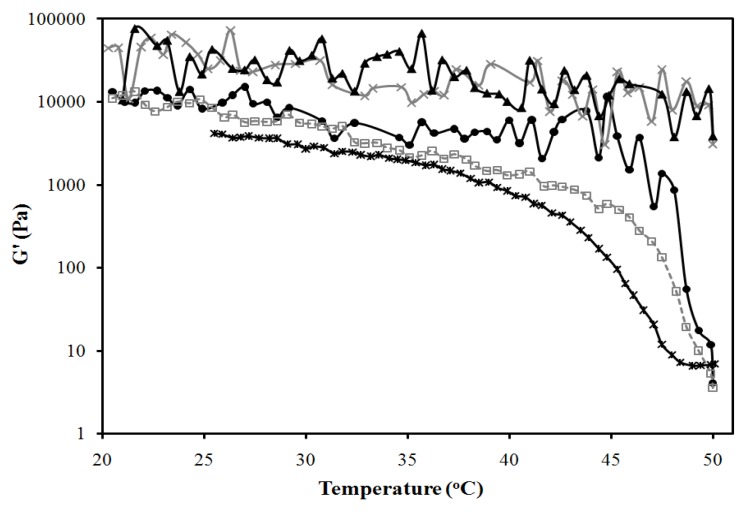
Effects of formulation components on elastic modulus of 20% gelatin gels: (●) control (20% gelatin in 0.9% NaCl solution) [log(G’,Pa) = 4.7(±0.3) − 0.027(±0.009)T(°C), (R^2^ = 0.27)]; (−) control with 5% lecithin [log(G’,Pa) = 4.8(±0.3) − 0.019(±0.009)T(°C), (R^2^ = 0.20)]; (▲) control with 5% lecithin and 6% SMO [log(G’,Pa) = 4.9(±0.3) − 0.022(±0.009)T(°C), (R^2^ = 0.19)]; (□) control with 10% PEG-6-caprylic/capric glycerides and 4% decaglycerol monocaprylate/caprate [log(G’,Pa) = 4.67(±0.08) − 0.058(±0.002)T(°C), (R^2^ = 0.95)]; (*) control with 5% lecithin, 6% SMO, 10% PEG-6-caprylic/capric glycerides and 4% decaglycerol monocaprylate/caprate [log(G’,Pa) = 5.28(±0.09) − 0.063(±0.003)T(°C), (R^2^ = 0.95)]. The equations in brackets represent a linear regression between log(G’) and T (from 25 to 40 °C), and the ±deviations in parentheses represent the standard deviation of the regression parameters (Excel 2010 data analysis pack).

Lecithin-linker gels (not MBGs) were prepared using selected mixtures of lecithin and linkers at the ratios corresponding to the 6% SMO formulation of Figure 1. Figure 11 presents the elastic modulus of these formulations as a function of temperature. Liphophilic components (lecitihin, SMO) slightly increased the G’ value of the gelatin gel—although the statistical regressions of Figure 11 indicate no significant difference for temperatures ranging from 25 °C to 40 °C—particularly at 50 °C. This observation is consistent with the fact that lecithin and SMO can also produce organogels on their own [34]. On the other hand, introducing the hydrophilic additives (linkers) PEG-6-caprylic/capric glycerides and decaglycerol monocaprylate/caprate decreased the G’ of the gelatin gel and its transition temperature from 50 °C to values close to 45 °C. This observation suggests that the ethylene glycol and glycerol groups of the hydrophilic linkers interfere with the self-assembly of the collagen strands during gelation, thus reducing the strength of the resulting gel [70].

### 3.3. *In Vitro* Transport Studies

The 20% gelatin MBG prepared with 6% SMO (Figure 3a) was evaluated as a delivery vehicle to load lidocaine in the skin, and use the skin as a lidocaine reservoir (*in situ* patch) for extended release. Using a permeable synthetic membrane, instead of skin, the gel itself was evaluated as a reservoir for the extended release of lidocaine. The corresponding 6% SMO μE was used as a control to compare the transport of lidocaine in both scenarios. 

#### 3.3.1. Transport in the Skin

The total mass of lidocaine, loaded in the skin from the μE (control), and the MBG were determined by adding the mass of lidocaine recovered from methanol extraction of the pig ear skin at the end of the experiment and the mass of lidocaine recovered from the receiver solutions at different times. Slightly less lidocaine (0.8 ± 0.3 mg/cm^2^) was loaded in the skin from the MBG than from the μE (1.3 ± 0.5 mg/cm^2^). The loading of lidocaine in pig skin samples from water, IPM and from lecithin-linker μEs has been evaluated by Yuan *et al.* via a skin-donor partition coefficient (K_sd_) [30]. This K_sd_ parameter was obtained after fitting the cumulative lidocaine permeation data to a three-compartment (donor, skin, and receiver) transport model. The value of K_sd_ represents the partition ratio at equilibrium between the lidocaine concentration in the skin compartment and the lidocaine concentration in the donor compartment. The value of K_sd_ can be estimated using the concentrations in the donor and in the skin—obtained via mass balance—after 30 minutes of loading [30]. The estimated values of K_sd_ were 0.36 ± 0.14 for the MBG and 0.46 ± 0.17 for the μE, comparable to the K_sd_’s reported for oil–continuous (K_sd_ = 0.3) and water-continuous (K_sd_ = 0.4) lecithin-linker μEs prepared with 4% lecithin [30], which are close to the parent μE of the MBG. These values are substantially larger than the partition from IPM (K_sd_ ~ 0.1) and substantially lower than the partition from water (K_sd_ ~ 1.3) [30]. The observed order of K_sd_’s correlate with the hydrophilicity of the donor vehicle; since lidocaine is a lipophilic drug, its partitioning into skin would be higher in more hydrophilic donor vehicles

**Figure 12:**
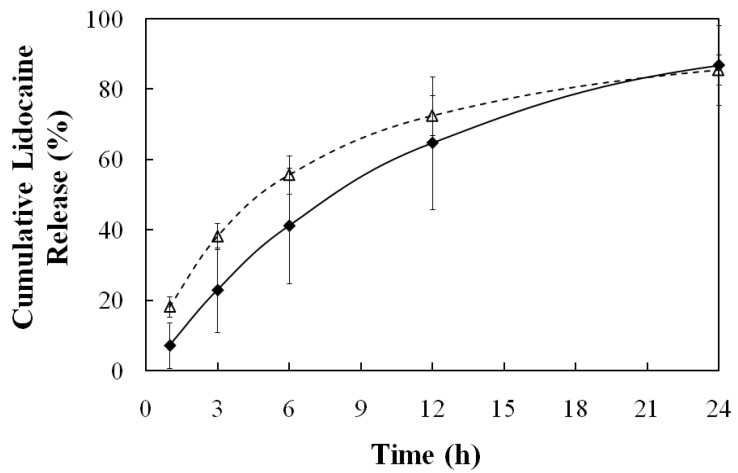
Release profile at room temperature (25 ± 1 °C) of lidocaine from pig skin after 30-minutes loading with: 20% gelatin MBG containing 3.1% lidocaine (♦), and gelatin-free, 6% SMO μE containing 3.9% lidocaine (∆). See Table 1 for additional μE composition details.

Figure 12 shows the lidocaine release profile for the MBG- and the μE-loaded skins. Both release profiles are similar, and akin to a first order release. The three compartment transport model of Yuan *et al.* can be simplified to a two-compartment case for the release of lidocaine from the skin to the receiver [23,30], that under sink conditions leads to a first order release model where the first order rate constant is k_sr_/h, k_sr_ being the skin-receiver transport coefficient (cm/hr) and h the thickness of the skin compartment (0.08cm). When the data of Figure 12 is plotted in the format of ln(1-fraction released) *vs.* time for the interval of 0–12 hrs for the μE system, k_sr_ = 9 ± 2 ∙ 10^−3^ cm/hr (R^2^ = 0.91 for the first order model), and for the interval of 0–24 hrs for the MBG system k_sr_ = 7 ± 4 ∙ 10^−3^ cm/hr (R^2^ = 0.99 for the first order model). Yuan *et al.* reported values of k_sr_ = 12∙10^−3^ cm/hr for water-continuous μEs and k_sr_ = 35 ∙ 10^−3^ cm/hr for oil-continuous μEs prepared with 4% lecithin, 12% SMO and a mixture of sodium octanoate and octanoic acid used as hydrophilic linkers [30]. The authors discussed that lower k_sr_ values were obtained with higher lecithin concentrations, and that this correlation might be associated with the penetration of lecithin, and possibly the linkers, into the skin [30]. It is possible that the low k_sr_ values of the MBG and the parent μE are associated with the relatively high concentration of lecithin and hydrophilic linkers. For extended release/extended action, however, lower k_sr_ values are desirable. These formulations offer the potential for longer lasting pain relief when compared to commercial lidocaine creams such as EMLA (emulsion containing 2.5 wt% lidocaine and 2.5 wt% prilocaine), whose action only lasts between 2 and 4 hours [71,72]. Unfortunately, attempts to establish direct comparisons between lecithin-linker μEs and the EMLA cream resulted in inconclusive results as the EMLA cream could not be homogeneously applied in the MPD device. 

One important observation derived from the release profiles of Figure 12 is that, other than a minor reduction in lidocaine loading, the increase in viscosity for the MBG formula did not affect the release of lidocaine from the skin. These results support the initial hypothesis that the gelatin gel network should not affect the loading or release of drugs on the skin, but produce a viscosity suitable for topical/transdermal applications.

#### 3.3.2. Transmembrane Transport

In order to produce a simplified model for periocular transport, skin was replaced by a cellulose acetate membrane with a molecular weight cut off (MWCO) of 100 kDa, which corresponds to an approximate pore size of 10 nm. The use of synthetic ultrafiltration membranes, particularly for scleral transport, has been reported in the literature [73,74,75]. Various ranges of equivalent pore size of the sclera have been reported in the literature, but for paracellular transport the pore size has been estimated to be in the order of 2–4 nm, although pore sizes approaching 10 nm have been proposed as well [16,76,77].

**Figure 13:**
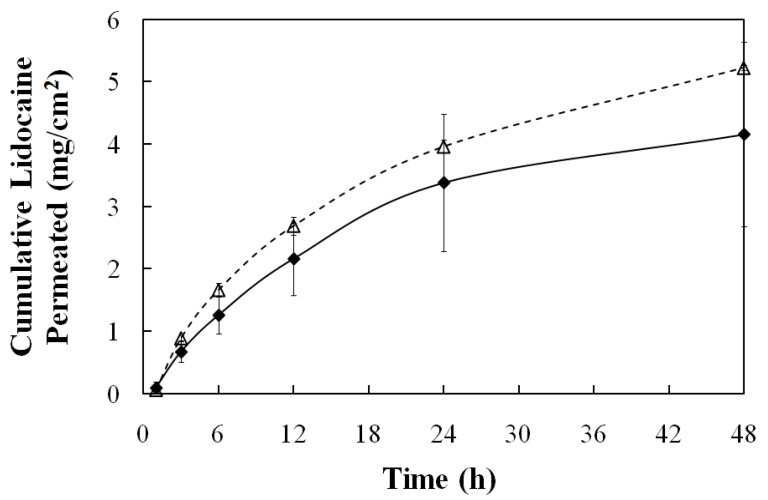
Permeation profile of lidocaine at 34 ± 1 °C through 100 kDa Molecular Weight Cut-Off (MWCO) acetate membrane from gelatin MBG containing 3.1% lidocaine (dose 6.1mg/cm^2^) (♦) and from lidocaine-loaded, gelatin-free, 6% SMO μE containing 3.9% lidocaine (dose 7.6 mg/cm^2^) (∆). See Table 1 for additional μE composition details.

Figure 13 presents the accumulated mass of lidocain permeated (per unit of area) through the acetate membrane as a function of time. Assuming that the membrane does not accumulate drug or microemulsion components, then a simple permeation model can be used to interpret the data of Figure 13. Assuming sink conditions, the transport equation based on the permeability coefficient can be simplified to: dC_d_/dt = −(k_p_*A/V))*C_d_ where C_d_ is the concentration of lidocaine in the donor compartment, k_p_ is the transmembrane permeability of lidocaine, A is the area of the membrane (0.256 cm^2^), and V is the volume of the donor compartment. Applying a simple mass balance to the data of Figure 13 and the initial concentration of lidocaine in the donor (C_do_), the value of C_d_ can be estimated as a function of time. A plot of ln(C_d_/C_do_) versus time should produce a straight line with a slope −(k_p_ A/V). This was the case when the data between 0 and 24 hrs was used. For the case of the MBG, the value of k_p _was 6 ± 1 ∙ 10^−3^ cm/hr and for the μE was 6.3 ± 0.4 ∙ 10^−3^ cm/hr. These values of k_p_ are close to the transdermal permeability of lidocaine, k_p_ ~ 20 ∙ 10^−3^ cm/hr [22], and to the permeability of other organic molecules through the sclera (k_p_ ~ 6 to 20 ∙ 10^−3^ cm/hr) [78].

This transmembrane transport study showed that the addition of gelatin did not affect the transport of lidocaine in conditions that simulate periocular drug delivery. The fact that similar lidocaine loadings (partition) in the skin were obtained with the μE and the MBG, further support the idea that the introduction of gelatin does not affect the transport of the drug. 

The permeation of lidocaine through the MBG can be interpreted using obstruction-diffusion models applied to heterogeneous hydrogels [79]. According to a simplified form of the equation of Amsden [79], for small drug molecules (e.g., lidocaine) in gel networks with relatively large distance between fibers, the diffusivity in the gel ≈ diffusivity in the solvent * exp(-(π/4)(thickness of the fiber/distance between fibers)^2^). Considering the distance between the fibers, and the thickness of the fibers in Figure 4 and Figure 5, and the expression of Amsden, one simply concludes that the fibers are too loosely packed to interfere with the diffusion of molecules through the gel. However, the packing is strong enough to increase the viscosity of the μE. 

## 4. Conclusions

This work introduced the formulation of alcohol-free, lecithin-based, gelatine MBGs. This linker formulation, found to be biocompatible in previous work, used lecithin as the main surfactant, SMO as a lipophilic additive (linker), and a mixture of PEG-6-caprylic/capric glycerides and decaglycerol monocaprylate/caprate as hydrophilic additives (linkers). It was found that in order to prepare clear MBGs (*i.e.*, absence of an emulsified phase in the final gel), it was important to ensure that the parent μE was a single phase at room temperature before introducing gelatin.

When gelatin was added to bicontinuous lecithin (nonionic) μEs, the results suggest that some of the water initially solubilised in the μE was used to produce a dispersed network of gelatin fibers embedded in an oil-rich bicontinuous μE. This observation contrasts with previous findings for anionic (AOT) MBGs where the addition of gelatin produced minor changes in the morphology of the μE. 

The elastic modulus (G’) of the MBGs increased with increasing water content or decreasing oil content. In lecithin-linker organogels, the addition of lipophilic components, lecithin and SMO, slightly increased the elastic modulus at high temperature, while the addition of hydrophilic additives reduced the elastic modulus of the gelatin gel at high temperature. 

When a single phase μE system was used as the “parent” μE for the MBG, a clear gel with a viscosity suitable for topical applications was obtained. This MBG produced comparable, although slightly lower, lidocaine loading and release from skin than the parent μE. 
